# The hidden wounds of childhood trauma

**DOI:** 10.1080/20008198.2017.1375840

**Published:** 2017-10-17

**Authors:** Andrea Danese, Anne-Laura van Harmelen

**Affiliations:** ^a^ MRC Social, Genetic and Developmental Psychiatry Centre, Institute of Psychiatry, Psychology and Neuroscience, King’s College London, London, UK; ^b^ Department of Child and Adolescent Psychiatry, Institute of Psychiatry, Psychology and Neuroscience, King’s College London, London, UK; ^c^ National and Specialist CAMHS Trauma and Anxiety Clinic, South London and Maudsley NHS Foundation Trust, London, UK andrea.danese@kcl.ac.uk; ^d^ Department of Psychiatry, University of Cambridge, Cambridge, UK

Although the role of intense psychological distress in psychopathology has been recognized for centuries, the metaphorical use of the word ‘trauma’ to indicate intense psychological distress only became popular in the midst of the industrial revolution (Danese & Baldwin, 2017). The introduction of the steam engine and the spread of railways led to a sharp increase in train accidents. Strikingly, many accidents involved previously healthy individuals who developed mental illness even in the absence of physical injury – the so-called ‘railway spine’. These unusual observations triggered a heated debate. On the one side, there were proponents of organic explanations (e.g. Oppenheim), who thought train accidents could have caused yet undetectable brain injuries leading to psychopathology. On the other side, there were proponents of functional explanations (e.g. Charcot), who thought that the mental/intra-psychic representation of the accidents – so-called ‘psychological’ trauma – could lead to psychopathology.

Neurobiological research is reconciling this conflict by suggesting that psychological trauma, and particularly childhood psychological trauma, can trigger the same physiological response as physical trauma. The biological plausibility of this theory is supported by experimental and observational studies showing that psychological stress can trigger an immune response or, more precisely, an inflammatory response (Steptoe, Hamer, & Chida, 2007), presumably with the aim of preparing the body to face potential physical injury (Segerstrom & Miller, 2004). Psychological stress can induce activation of the amygdala and consequently of the sympathetic nervous system, which in turn triggers activation of immune cells and the inflammatory response (Bierhaus et al., 2003). Inhibitory systems, namely the hypothalamic-pituitary-adrenal (HPA) axis and the parasympathetic nervous system, generally keep the inflammatory response at bay preventing its chronic activation.

Psychological traumas occurring early in life may, however, have long-term effects on the immune system. Of note, individuals with a history of childhood trauma, such as childhood maltreatment, show greater amygdala reactivity to emotional stimuli (van Harmelen et al., 2013) and, thus, may more often experience activation of the inflammatory response. Furthermore, they show reduced HPA axis signalling (Heim, Newport, Mletzko, Miller, & Nemeroff, 2008), and, because of the impairment in this inhibitory pathway, they may also show chronic elevation in inflammation levels. We reported initial evidence of association between childhood maltreatment and high levels of several inflammation biomarkers in 1000 members of the Dunedin Multidisciplinary Health and Development Study, who were followed up from birth to age 32 years (Danese, Pariante, Caspi, Taylor, & Poulton, 2007). This association has since been replicated in more than two dozen human studies. However, observational human studies cannot conclusively established causality because of potential confounding by unmeasured factors. Therefore, it is important to test whether early life stress has consistent effects on inflammation in animal models, where the experimental allocation to stress enables researchers to make causal inference. For example, experimental research in non-human primates showed that peer rearing led to differential expression of genes in immune cells compared to maternal rearing, with increase in the expression of genes related to inflammatory pathways (Cole et al., 2012). Furthermore, research in rodents showed that maternal separation led to increase in both blood inflammation biomarkers and in the density of the brain resident immune cells, the microglia (Delpech et al., 2016). Therefore, early life stress can impact not only on peripheral inflammation levels but also on brain inflammation.

The link between childhood trauma and inflammation may help us understand the enduring effects of childhood trauma on later mental and physical health (Agnew-Blais & Danese, 2016; Danese & Tan, 2013; Nanni, Uher, & Danese, 2012). Inflammation can affect the risk for psychopathology by altering the metabolism of key neurotransmitters including monoamines and glutamate; in addition, when acting in early life, inflammation can also impact on brain development and influence the reactivity of the HPA axis and microglia to later stressors (Danese & Lewis, 2017). Inflammation also affects risk for cardiovascular disease and type 2 diabetes by influencing atherosclerosis progression and insulin sensitivity (Danese & McEwen, 2012). Supporting the role of inflammation in mediating the association between childhood trauma and later disease, experimental research in rodents showed that administration of anti-inflammatory medications can buffer cognitive impairment after early life stress (Brenhouse & Andersen, 2011). However, more research is needed to directly test mediation processes in humans (Danese, 2014).

If inflammation explains the link between childhood trauma and later disease, research in this area can have important clinical implications for secondary and tertiary prevention. Secondary prevention refers to the possibility of reducing the risk of onset of clinical conditions after trauma exposure. If inflammation mediates these effects, then reduction in inflammation would decrease risk of illness. Strategies to reduce inflammation in the context of secondary prevention may include broad interventions targeting unhealthy behaviours including over-eating, lack of physical activity, substance abuse and poor sleep (Danese & Baldwin, 2017; Danese & Lewis, 2017). Tertiary prevention refers to the possibility of reducing the severity of clinical conditions in affected individuals with a history of childhood trauma. This is equally important given the evidence that adults with a history of childhood trauma have greater risk of showing unfavourable clinical characteristics, course of illness and treatment response, for example in the context of affective disorders (Agnew-Blais & Danese, 2016; Nanni et al., 2012). If inflammation contributes to these effects, then treatments that reduce inflammation would decrease the severity of trauma-related illness. Strategies to reduce inflammation in the context of tertiary prevention may include anti-inflammatory medications, which for example have shown some beneficial effects in treatment-resistant depression related to high inflammation (Raison et al., 2013).

In sum, further understanding of the biological processes that translate the exposure to childhood trauma into biological risk for disease (biological embedding) may point to innovative strategies for the treatment of trauma-related psychopathology and medical conditions (Lanius & Olff, 2017).
